# Supplementary material for: Micro-thermocouple on nano-membrane: thermometer for nanoscale measurements
Source: Sci Rep. 2018 Apr 20;8:6324. doi: 10.1038/s41598-018-24583-w (PMC5910443; doi:10.1038/s41598-018-24583-w)
Supplement: Supplementary file 1 — Supplementary Information [file 41598_2018_24583_MOESM1_ESM.pdf]

Supplementary material: Micro-thermocouple on nano-membrane: thermometer for nanoscale measurements

Armandas Balčytis, Meguya Ryu, Saulius Juodkazis, and Junko Morikawa

Transient thermal analysis for the sensing system is done by using quadrupole method [1-4]. The system is simplified by considering parallel connection of 1D heat transfer segments. The heat induced by the electron beam irradiation at distance x [m] from the Au-Ni hot junction of the thermocouple is dissipated through three different passes (Fig1 (a)). Figure 1 (b) shows equivalent electrical circuit of this model with four different transfer matrixes corresponding to the SiN substrate and Au/Ni wire (the high thermal conductivity of Au is the main channel of heat dissipation from the Au/Ni thermocouple). With this model, the analytical solution of the phase and amplitude of the signal measured by the thermocouple at the detection point can be calculated using the Laplace transform of temperature, $\theta_0 = \mathbf{L}\{T\}$ (**L** denotes the Laplace transform) defined by the heat conduction equation [4]:

$$\theta_0 = \{A_1(x) + YB_1(x)\}X\phi_0,$$

where X and Y can be expressed as follows,

$$X = -\left\{\frac{A_1(d_0)+Z_0C_1(d_0)}{B_1(d_0)+Z_0D_1(d_0)} + Y\right\},$$
$$Y = -\frac{(M_1+M_2)A_1(x)+M_1M_2C_1(x)}{(M_1+M_2)B_1(x)+M_1M_2D_1(x)},$$
$$M_1 = \frac{B_1(d_0-x)+Z_0D_1(d_0-x)}{A_1(d_0-x)+Z_0C_1(d_0-x)},$$
$$M_2 = \frac{B_2+Z_0D_2}{A_2+Z_0C_2},$$

where d_0 is the half width of the membrane aperture size and x is the distance between electron beam heating position and detection point (the hot junction of thermocouple). Here, A_n B_n C_n D_n correspond to the components of the transfer matrix for each material [1]. Each component can be written as follows,

$$A = D = \cosh\left(\sqrt{\frac{j\omega}{a}}d\right),$$
$$B = \frac{\sinh\left(\sqrt{\frac{j\omega}{a}}d\right)}{e\sqrt{j\omega}},$$
$$C = e\sqrt{j\omega}\sinh\left(\sqrt{\frac{j\omega}{a}}d\right),$$

where, ω is the angular frequency of the induced thermal signal [rad], a is the thermal diffusivity of the sample [m^2/s], d is the thickness of the sample [m], and e is the thermal effusivity of the sample [$JK^{-1}m^{-2}s^{-1/2}$].

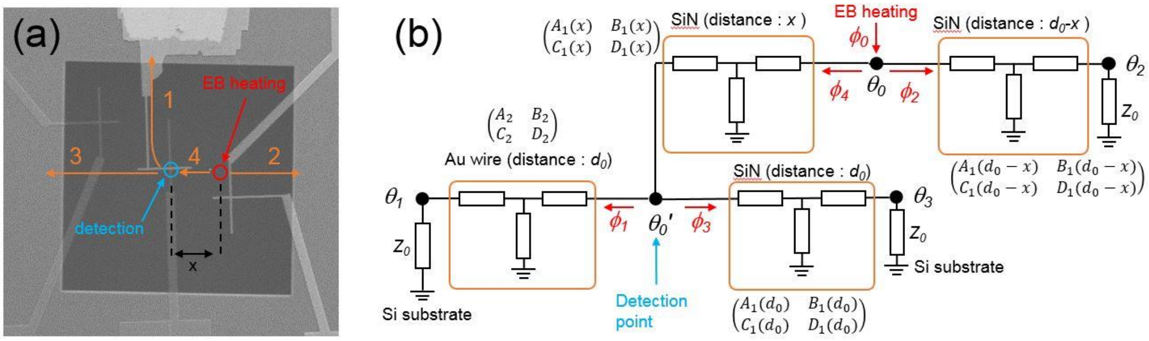

Fig. 1. (a) SiN nano-membrane on Si substrate with Au/Ni thermocouple in the middle. (b) Equivalent electrical scheme of heat flow calculations using quadrupole method [4] (see notations in the text above). The heat dissipation channels are marked in (a): 1 – from the thermocouple to the Si substrate (of a constant temperature) will prevail via Au lead, 2 – heat flow from heating point through the SiN nano-membrane, 3 – from the heating point through the nano-membrane till the thermocouple (path 4 in (b)) and then to the Si substrate.

We considered that the detected signal is a combination of the direct electron beam injection and the temperature rise. The temperature signal is calculated by using the above defined analytical solution using the following values of thermophysical properties of materials (Table 1).

Table 1. Material properties used in calculations.

Material	a (m^2s^{-1})	λ ($Wm^{-1}K^{-1}$)	C_p ($Jg^{-1}K^{-1}$)	ρ (kg/m^3)
SiN	1.3×10^{-6}		0.7113	3150
Au	1.2×10^{-4}	315	0.1290	19300
Si		156	0.7150	2329

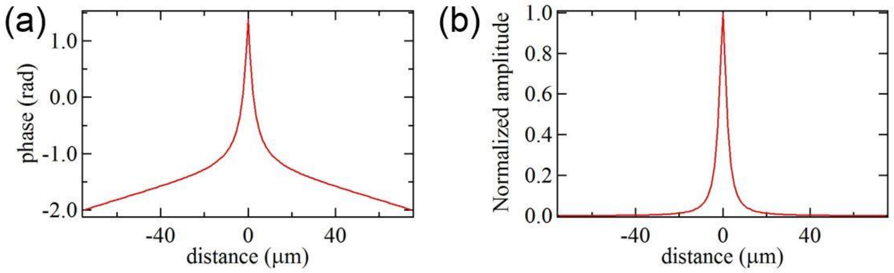

Fig. 2. Calculation result of the (a) phase and (b) amplitude of the temperature wave signal detected at the hot junction of the sensor as a function of the distance between the junction and the heating point (x). Frequency of the temperature wave is same as experimental condition (27 Hz), and the half width of the aperture is set to 125 μm. Values in table 1 is used for the thermophysical properties of each material.

The result of the phase and amplitude signal at the used 27 Hz modulation of the electron beam is shown in Fig. 2. The phase slope at larger distances becomes linear and correspond to the thermal diffusivity of SiN as it is expected. Figure 3 shows modelling results for several frequencies of e-beam irradiation. As the frequency becomes high, the thermal diffusion length $\mu = \sqrt{\frac{a}{\pi f}}$ becomes short. In this case, the Au wire connection to the semi-infinite layer of Si is strongly influence attenuation of the phase and amplitude at the centre (x = 0) at high frequency (a spatially limited

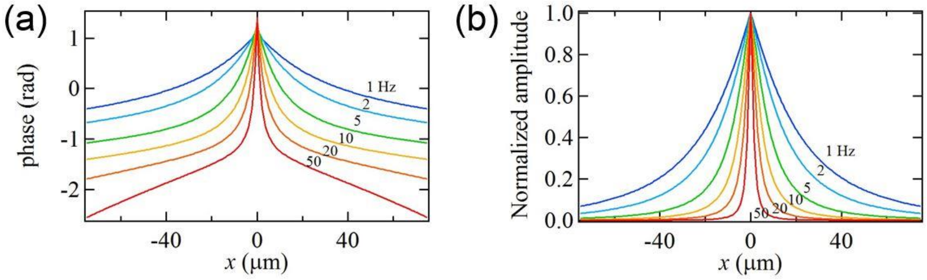

Fig. 3. Calculation result of the (a) phase and (b) amplitude of the temperature wave signal detected at the hot junction of the sensor as a function of the distance between the junction and the heating point (x) in the case of different frequency (1, 2, 5, 10, 20, and 50 Hz) of induced temperature wave.

case). The high frequency temperature wave signal has an advantage of spatial decoupling of Au wire, SiN membrane and Si substrate, but for the practical experiment, the absolute value of the signal become small and decrease S/N ratio of detection.

References

1. A. Degiovanni, Conduction dans un “mur” multicouche avec sources : extension de la notion de quadripôle, Int.J.Heat.Mass.Transfer. Vol. 3, 553 - 557, 1988.
2. J.C.Batsale, D.Maillet, A.Degiovanni, Extension de la méthode des quadripôles thermiques à l'aide de transformations intégrales-Application au défaut plan bidimensionnel Int.J.Heat.Mass.Transfer,Vol. 37, 111-127, 1994.
3. D. Maillet, S. André, J.C. Batsale, A. Degiovanni, C. Moyne, “Thermal quadrupoles : Solving the heat equation through integral transforms”, Wiley, London, 2000.
4. J.Pailhes C.Pradere J.-L.Battaglia J.Toutain A.Kusiak A.W.Aregba J.-C.Batsale, Thermal quadrupole method with internal heat sources, International Journal of Thermal Sciences 53, 49-55 2012.
